# Combined Microwave Ablation and Osteosynthesis for Long Bone Metastases

**DOI:** 10.3390/medicina57080825

**Published:** 2021-08-16

**Authors:** Claudio Pusceddu, Giuseppe Dessì, Luca Melis, Alessandro Fancellu, Giuseppe Ruggiu, Paolo Sailis, Stefano Congia, Daniele Derudas, Riccardo Cau, Ignazio Senis, Nicola Ballicu, Luca Saba

**Affiliations:** 1 Department of Oncological and Interventional Radiology, Businco Hospital, 09121 Cagliari, Italy; clapusceddu@gmail.com (C.P.); nicolaballicu77@gmail.com (N.B.); 2 Department of Orthopedics and Traumatology, Brotzu Hospital, 09121 Cagliari, Italy; giuse.dessi@tiscali.it (G.D.); giusepperuggiu@aob.it (G.R.); paolo.sailis@gmail.com (P.S.); stefano.congia@tiscali.it (S.C.); 3 Nuclear Medicine Department, Businco Hospital, 09121 Cagliari, Italy; doclucamelis@tiscali.it; 4 Department of Medical, Surgical and Experimental Sciences, University of Sassari, 07100 Sassari, Italy; 5 Department of Hematology, Businco Hospital, 09121 Cagliari, Italy; danielederudas@tiscali.it; 6 Department of Radiology, Policlinico Universitario, 09121 Cagliari, Italy; riccardocau00@gmail.com (R.C.); ignazio.senis@gmail.com (I.S.); lucasabamd@gmail.com (L.S.)

**Keywords:** microwave ablation, bone metastases, osteosynthesis

## Abstract

*Background and Objectives:* The purpose of this study was to evaluate the feasibility, safety and efficacy of microwave ablation (MWA) in combination with open surgery nail positioning for the treatment of fractures or impending fractures of long bone metastases. *Material and Methods:* Eleven patients (four men, seven women) with painful bone metastases of the humerus, femur or tibia with non-displaced fractures (one case) or impending fractures (10 cases) underwent open MWA in combination with osteosynthesis by locked nail positioning. Pain intensity was measured using a VAS score before and after treatment. CT or MRI were acquired at one month before and 1, 3, 6, 12 and 18 months after treatment. *Results:* All procedures were successfully completed without major complications. The level of pain was significantly reduced one month after treatment. For the patients with humerus metastases, the complete recovery of arm use took 8 weeks, while for the patients with femoral metastases the complete recovery of walking capacity took 11 weeks. The VAS score ranged from 7 (4–9) before treatment to 1.5 (0–2.5) after treatment. During a mid-term follow-up of 18 months (range 4–29 months), none of the patients showed tumor relapse or new fractures in the treated site. Two patients died due to tumor disease progression. *Conclusion:* Results of this preliminary study suggest that combined MWA and surgical osteosynthesis with locked nails is a safe and effective treatment for pathological fractures or malignant impending fractures of long bone metastases of the humerus, femur and tibia. Further analyses with larger cohorts are warranted to confirm these findings.

## 1. Introduction

Long bones, in particular the femur, are the target of metastases frequently characterized by the occurrence of pain and pathological fractures [1]. About 60% of all painful impending or actual pathologic fractures occur in this area, causing substantial disability and restriction in ambulation [2,3]. Currently, there is a growing interest in the management of patients with femur metastases due to the increasing life expectancy of patients and the opportunity to maintain good function and quality of life [4].

Notably, most of the metastatic lesions arising from breast, kidney or thyroid cancer, as well as from hematologic malignancies, are lytic and this subtype of metastases is associated with an increased risk of pathologic fracture [2,3]. Treatment of those metastatic lesions is warranted to reduce the risk of fracture and obtain pain control. The classic surgical approach comprises multiple approaches, including endoprosthetic replacement (EPR), proximal femoral replacements (PFRs) and intramedullary nail (IMN) fracture fixation [5,6].

New approaches have been introduced recently to treat long bone metastases, such as tumor ablation techniques which are gaining growing evidence of efficacy [7]. Tumor ablation is a minimally invasive technique where thermal energy is used to heat or cool tissue to cytotoxic levels (less than −40 °C or more than 60 °C). There are different types of technologies, based on different physics methods, that can carry out tumor ablation. Among them, microwave ablation (MWA) is a new and promising approach [8]. 

The purpose of this study was to evaluate the feasibility, safety and efficacy of MWA in combination with open surgery nail positioning for the treatment of fracture or impending fractures of long bone metastases.

## 2. Materials and Methods

### 2.1. Study Design and Patient Population 

In this preliminary analysis, 11 patients (4 men and 7 women, mean age 65 (52–81 years)) with painful bone metastases of the humerus (4 cases), femur (6 cases) and tibia (1 case) were treated with MWA in combination with osteosynthesis by locked nail positioning. Informed consent to perform the procedure was obtained from all patients included in the study. Before the procedure, the board (oncologist, interventional radiologists, radiotherapist, pathology and orthopedic surgeons) assessed the condition of the patients and approved the surgical approach with consensus. All patients underwent analysis of the neoplasm with CT and/or MR imaging and all those cases with an extramedullary tumor component were excluded from these procedures. The diagnosis was confirmed in all patients by performing histopathologic examination. Assessment of the pain was quantified by using the analogical VAS pain scale 4 weeks before the treatment and 1, 3, 6, 12 and 18 months after treatment. Mortality and/or re-occurrence of pathology was recorded in the follow-up.

### 2.2. MWA Technique

**Figure 1:**
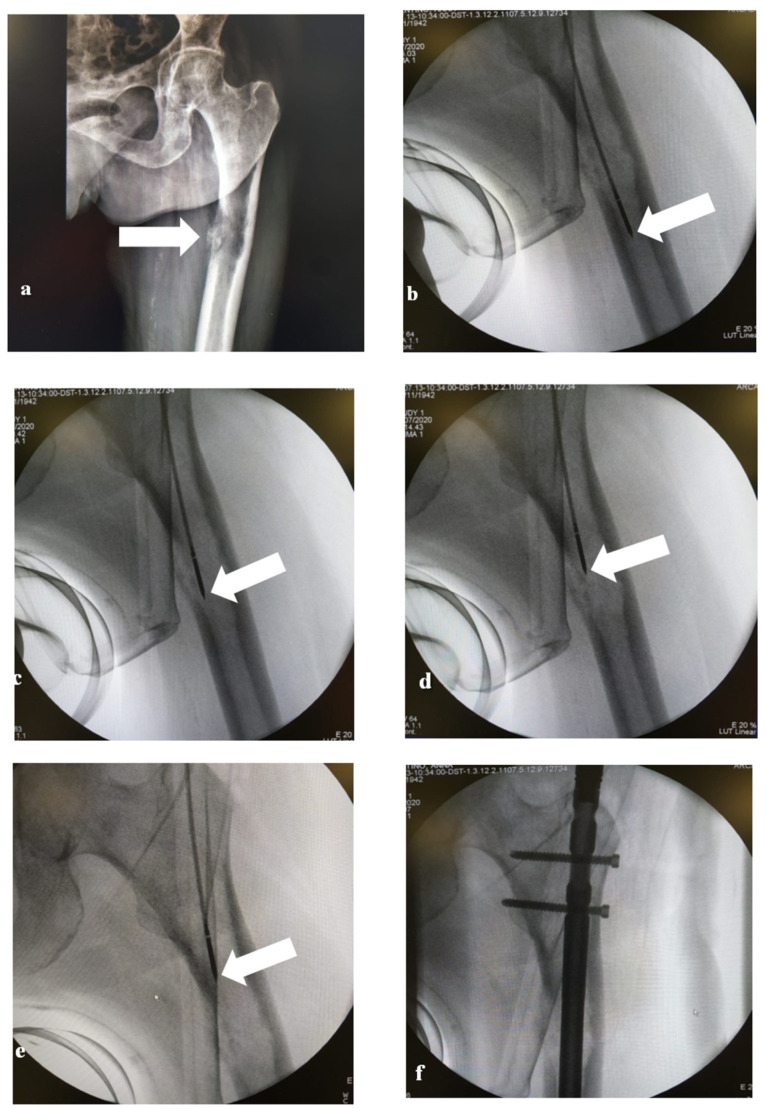
A 54-year-old female patient with a left femur metastasis from breast cancer. The X-ray (**a**) shows a lytic lesion of the left femur with erosion of the medial cortical profile. The C-Arm images (**b**–**e**) show 4 different positions of the MW antenna from distal to proximal edge of the lytic lesion. The C-Arm image (**f**) shows the presence of the endoprosthetic replacement.

All the thermal ablation procedures were performed by using a 2.45 GHz microwave generator (AMICA-GEN; HS Hospital Service, Aprilia, Italy) with energy delivered via 14-gauge mini-choked, water-cooled interstitial antennae (HS AMICA; HS Hospital Service). The antennae had different lengths, from 15 to 20 cm used in the proximal diaphyseal lesions and 27 cm to ablate the more distal ones. Prior to the ablative treatment, the extent of the intramedullary tumor was evaluated with preoperative axial CT or MR scans in all cases. All studies were completed by sagittal and coronal electronic reconstruction planes necessary to compare the reconstructed images with the fluoroscopic images of the bone segment part to be treated. Based on the longitudinal extension and diameter of the epiphyseal or diaphyseal tract at the level of the tumoral tissue to be treated, we planned the power level and exposure time in order to obtain a coagulative area non-superior to the maximum diameter of the bone in that section. This approach was necessary to avoid overheating of the non-target anatomical structures around the bone, particularly the nerves. Once the access to the medullary canal was surgically prepared, and the biopsy performed, the antenna was inserted with the tip of the needle positioned at the most caudal edge of the lesion (Figure 1). The manufacturer’s treatment specifications were therefore adopted by applying an adequate power level and exposure time. In lesions 3 cm or less, a single application was considered able to obtain complete tumor necrosis. In larger lesions, the antenna was withdrawn by about 3 cm and, after fluoroscopic confirmation, the treatment was repeated until all of the lesion was covered. It took from 1 placement in minor lesions to 5 placements in major lesions. In a patient with a fracture, reduction and alignment of the diaphyseal canal were necessary before performing the insertion of the antenna and ablation of the tumor.

### 2.3. Surgical Technique

**Figure 2:**
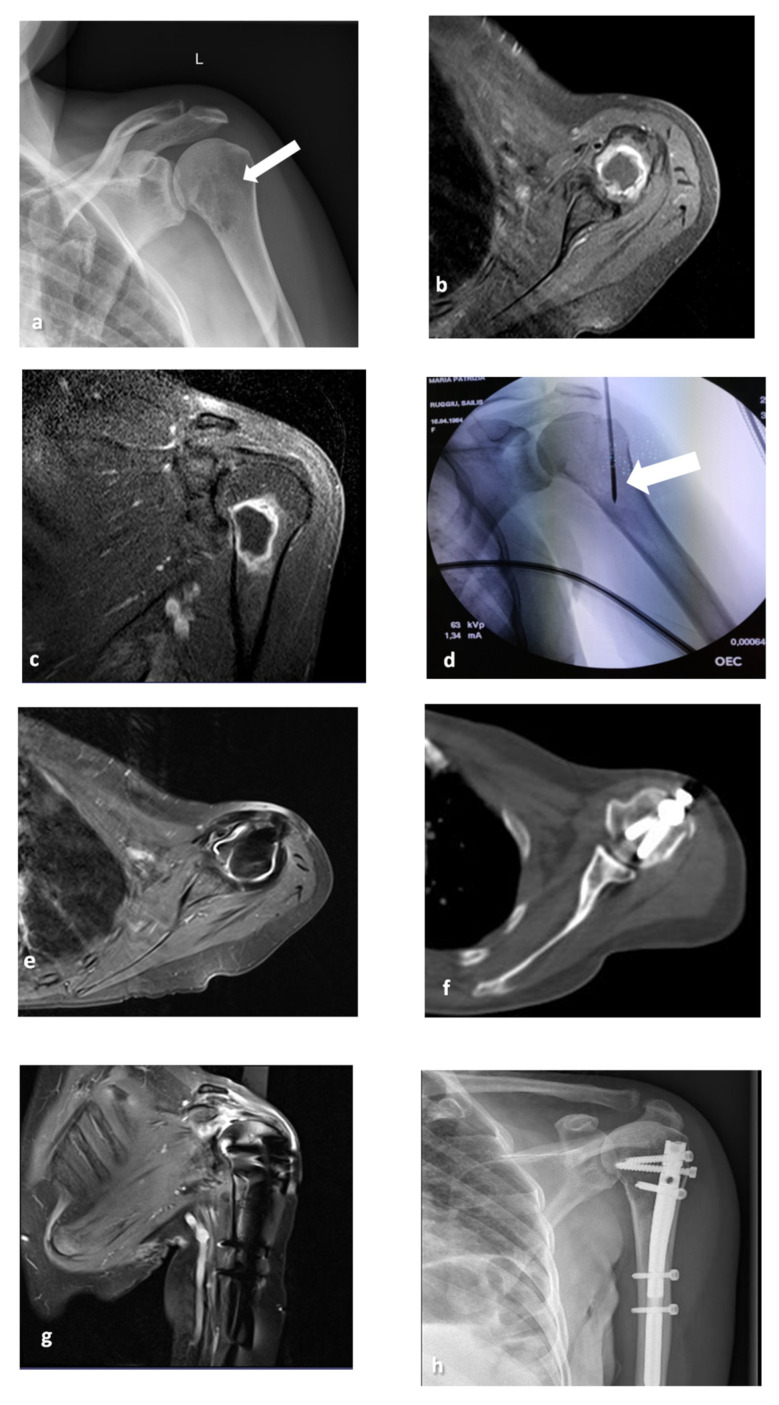
A 56-year-old female patient with a left humerus metastasis from breast cancer (**a**–**d**). Coronal X-ray shows a lytic lesion in the superior portion of the humerus (arrow). Axial and coronal T1-weighted MR scans with fat suppression after gadolinium infusion (**b**,**c**) show the lytic lesion surrounded by a rim of enhancement. The coronal C-Arm image (**d**) shows the MW antenna positioned in the lesion (arrow). After 1 year of follow-up, axial and coronal MRI (**e**–**g**), axial CT (**f**) and X-ray (**h**) show the presence of the endoprosthetic replacement and no local relapse in the treated site.

**Figure 3:**
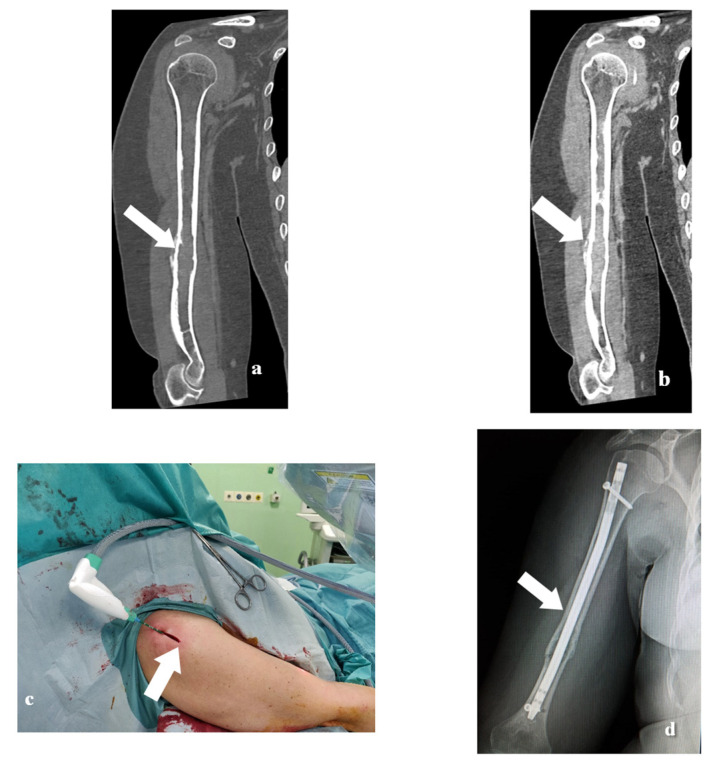
A 56-year-old female patient with a right humerus metastasis from breast cancer (**a**–**d**). Coronal CT with bone window (**a**) and soft tissue window (**b**) show the presence of a lytic lesion in the middle portion of the humerus. The panel (**c**) shows the treatment whereas the panel (**d**) shows the presence of the endoprosthetic replacement.

Impending fractures of the femur, tibia and humeral shaft were all treated with antegrade locking long nails. Preoperative antibiotic prophylaxis (cefazolin 2 intravenously) was infused and surgical sterile fields were prepared. The approach firstly involved creating a nail entry portal in the proximal tibia, femur and or humerus. This allowed performing the MWA procedure inside the medullary canal and, right after, fixing the impending fracture with an intramedullary device. We used the trans-deltoid with supraspinatus split approach for the humerus, infrapatellar through patellar tendon for the tibia and tip of great trochanter approach for the femur. All the procedures required the aid of a C-Arm. In each case, a guide wire was inserted through the bone shaft and when the medullary canal was narrow, reaming it was unavoidable and was made 1.5 mm larger than the nail diameter. Once the nail was inserted and the guide wire removed, we increased device stability by introducing blocking screws in multiple proximal and distal holes of the nail. An end cap was positioned at the tip of the nail; the proximal surgical wound (4–5 cm) used for the nail entry point and incisions (1–2 cm) used for screw insertion were closed in layers and secured with dressings. Antithrombotic prophylaxis was recommended for at least 30 days (Figure 2 and Figure 3). From the first day after surgery, patients were encouraged to get up from the bed and fully recover all previous functional daily autonomies without restrictions.

### 2.4. Statistical Analysis

The normality of each continuous variable group was tested using a Kolmogorov–Smirnov *Z* test. Continuous data were described as mean ± SD and binary variables were summarized as counts (percentages). Wilcoxon analysis was performed to check the difference in VAS score before and after the treatment. Mean follow-up was calculated, as well as percentages of patients alive and of those who had local relapse after MWA + osteosynthesis treatment. A *p* value <0.05 was regarded to indicate statistical significance and all correlation values were calculated using a two-tailed significance level. R software (www.r-project.org, accessed on 9 April 2021) was employed for statistical analyses.

## 3. Results

Eleven patients (four men, seven women; mean age 65 years old with age range 52–81 years old) were analyzed for a total of 11 metastatic lesions (Table 1). The average size was 67.5 mm (range 30–165 mm). In the femur (six cases), the average size was 73 mm (range 30–165 mm), in the humerus (four cases) the average size was 63 mm (range 37–100 mm) whereas in the tibia the average size was 53 mm (more details are given in Table 1). The mean Mirels’ score was 8.1 (range 7 to 10). The metastases were located in the epiphysis or proximal and middle diaphysis with an intramedullary tumor component. No infection nor other major complications were observed after the treatment. Arm function and bone stability were optimal during the considered follow-up. During a mid-term follow-up of 18 months (from 4 to 29 months), nine patients (81.8%) were alive, although in two of them disease progression was observed. Two patients died after 7 and 12 months due to brain and liver metastases, respectively. One patient (9.1%; patient No.3) developed a tumor relapse near the site treated with MWA and osteosynthesis after 16 months. However, none of the patients showed tumor relapse or new fractures in the treated site.

**Figure 4:**
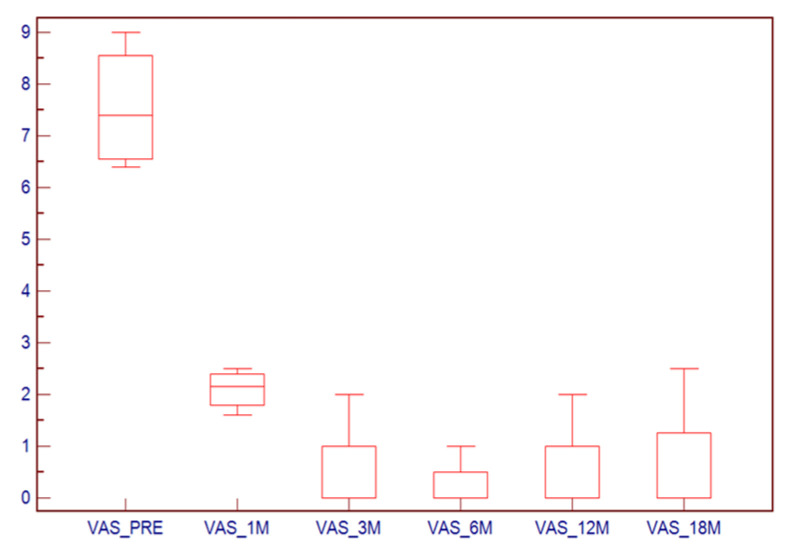
Box-plots of the VAS before treatment and in the follow-up at 1, 3, 6, 12 and 18 months.

The VAS score ranged from 7 (4–9) before treatment to 1.5 (0–2.5) after treatment. Wilcoxon analysis showed a statistically significant difference before and after treatment in all the follow-up checks (*p* < 0.001). In Figure 4 the box-plots are given. 

## 4. Discussion

Fractures or impending fractures of long bone metastases may lead to pain, pathological fractures, immobility or decreased arm functioning, and therefore the treatment of these conditions is extremely important [9]. Our preliminary study demonstrated the feasibility, safety and efficacy of MWA in combination with open surgery nail positioning in the multimodality treatment of long bone metastases.

The strategic treatment is determined by the level of the dissemination of the metastasis; in particular, it is possible to identify three main categories: solitary lesion, oligo-metastases (between two and four bone metastases) or diffuse [9]. In the case of some types of primary tumors, such as kidney cancer or bone sarcoma, the treatment can be curative [10].

In the 1990s, a Chinese team studied the application of surgical treatment of bone tumors in conjunction with microwave-induced hyperthermia with or without adjuvant immunotherapy [11,12] and found that the use of microwave hyperthermia and adjuvant immunotherapy in conjunction with surgical treatment can be considered a safe and well-tolerated procedure. However, this study was different from our analysis because the cohort was composed of mixed population with 79.4% of subjects with primary malignant neoplasms and 20.6% with benign ones and most of the tumors occurred on knee joints (72.6%), whereas in our study all the patients were treated for metastatic lesions. In 2012, Pap et al. [13] described the cases of three of 71 patients who were treated for impending pathologic fractures with intramedullary stabilization who developed soft tissue tumor recurrence along the surgical tract site postoperatively. Additionally, Miller et al. [14] in a retrospective study of 112 consecutive patients who underwent stabilization of the femur (81 cases), humerus (25 cases) and tibia (6 cases) reported that local tumor progression is one of the causes of surgical treatment failure after intramedullary nail implantation in long bone metastases.

One important point, other than the “technical potentiality” of this approach and the recurrence and survival information we have obtained, is the pain control quantified through the VAS analysis that showed an excellent control of the pain, with values from 7 pretreatment to 1.5 after treatment. After this type of procedure, it is possible to observe a re-increase in the VAS in the long-term follow-up [15,16], whereas in this case the long-distance pain control is good.

Notably, none of the patients of the present series received radiotherapy on bone metastases. Radiotherapy is performed primarily to relieve pain and prevent pathologic fractures. As witnessed from the VAS score and postoperative imaging, optimal pain control and bone stability were achieved with MWA plus osteosynthesis treatment. We believe that these results had a role in avoiding radiotherapy, although the small sample size prevents definitive conclusions.

We are aware that in this study there are some limitations. First, the small cohort size we have assessed; however, this study should be considered a preliminary analysis and a “proof of concept” where it is shown that it is possible to perform MWA in combination with open surgery nail positioning in the treatment of fracture or impending fractures of long bone metastases safely and with potentially very good results. Second, the metastases were from different types of primary tumors and this may have caused a bias in terms of homogeneity; it is possible to speculate that different histotypes could determine different results in terms of efficacy.

## 5. Conclusions

In conclusion, the results of this preliminary study suggest that combined MWA and surgical osteosynthesis with locked nails is a safe and effective treatment of pathological fractures or malignant impending fractures of long bone metastases of the humerus, femur and tibia. Further analyses with larger cohorts are warranted to confirm these findings and to better understand the role of MWA and surgical osteosynthesis in the multimodality treatment of metastatic disease with long bone involvement.

## Figures and Tables

**Table 1:** Demographics of patients with pathologic fractures or impending fracture of femur, humerus and tibia.

No.	Sex	Age	Primary Tumor	Localization of Bone Metastases	Size of Metastases (cm)	Fracture Type	Mirels’ Score	No. of MW Antenna Placements	MW Power/Time
1	M	68	Prostate	Humerus	10.0	IF	8	4	50 W 3 min
2	F	81	Breast	Femur	16.5	AF	-	5	60 W 3 min
3	F	57	Breast	Femur	3.0	IF	7	1	50 W 3 min
4	F	58	Breast	Femur	5.0	IF	8	3	50 W 3 min
5	F	56	Breast	Humerus	7.5	IF	7	4	50 W 3 min
6	F	56	Breast	Humerus	4.0	IF	8	2	50 W 3 min
7	M	60	NSCLC	Femur	5.4	IF	8	4	50 W 3 min
8	M	75	Kidney	Humerus	3.5	IF	8	2	50 W 3 min
9	F	54	Breast	Femur	7.5	IF	10	4	60 W 3 min
10	F	72	CRC	Tibia	5.3	IF	8	4	50 W 3 min
11	M	52	MM	Femur	6.9	IF	9	4	50 W 3 min

F, female; M, male; IF, impending fracture; AF, actual fracture; W, watts; CRC, colorectal cancer; NSCLC, non-small cell lung cancer; MM, multiple myeloma.

## Data Availability

Study data will be shared upon reasonable request.
